# Mechanical Properties of Ultra-High-Performance Concrete with Amorphous Alloy Fiber: Surface Modification by Silane Coupling Agent KH-550

**DOI:** 10.3390/ma17164037

**Published:** 2024-08-14

**Authors:** Dawei Wang, Runqing Liu, Song Wang, Xin Ma

**Affiliations:** School of Materials Science and Engineering, Shenyang Ligong University, Shenyang 110159, China; 13624007718@163.com (D.W.); liurunqing@sylu.edu.cn (R.L.); ws15169122715@163.com (S.W.)

**Keywords:** amorphous alloy fiber, high-performance concrete, surface modification, KH-550

## Abstract

Amorphous alloy fiber has the advantages of high tensile strength and high corrosion resistance compared with steel fiber, but its interfacial bonding with cement matrix is poor and requires surface modification treatment. In this study, the surface modification of amorphous alloy fiber was carried out by using silane coupling agent KH-550 solution, and its effect on the mechanical properties of ultra-high-performance concrete was investigated. The results showed that the amorphous alloy fibers modified with 15% concentration silane coupling agent KH-550 solution can effectively improve the mechanical properties of the ultra-high-performance concrete, where the interfacial bond strength with the cement matrix reached 3.29 MPa and the roughness reached 3.85. The compressive strength, flexural strength, tensile strength, and peak stress of the ultra-high-performance concrete mixed with modified amorphous alloy fibers could reach up to 133.6 MPa, 25.5 MPa, 8.32 MPa, and 114.26 MPa, respectively, which were 2.9%, 6.3%, 10.9%, and 4.3% higher than those of the ultra-high-performance concrete with unmodified amorphous alloy fibers. As the surface of the fiber was modified, its properties changed and the bonding effect with the cement matrix was better, which in turn improved the mechanical properties of the ultra-high-performance concrete.

## 1. Introduction

Ultra-high-performance concrete (UHPC) has the advantages of high strength, good toughness, and good durability. It has been widely used in highway bridges, construction projects, and other fields, and has become a hot spot in the research of cement-based materials at home and abroad [1,2]. As one of the important components of UHPC, the variety and dosage of fiber have an important influence on the performance of concrete [3,4,5]. At present, the fibers added to UHPC are mainly steel fibers, carbon fibers, polypropylene fibers, basalt fibers, etc. [6]. Among them, steel fiber is the most widely used, but there are problems such as poor corrosion resistance and high price [7,8]; carbon fiber has the characteristics of high strength, light weight, and good durability. However, carbon fiber is expensive, small in diameter, and hydrophobic on the surface so that the fiber cannot be uniformly dispersed in concrete, and it will also lead to the fluctuation of concrete resistivity, which greatly limits its engineering application [9,10]. Polypropylene fiber has the advantages of chemical corrosion resistance, good processability, and low price, and it has a significant effect on improving the plastic shrinkage cracking of concrete at low dosages, but there are also some shortcomings, such as poor strengthening and toughening effects on concrete [11,12]. Basalt fiber has the characteristics of corrosion resistance, high and low temperature resistance, green environmental protection, and high cost performance, but it has little effect on the compressive strength of concrete [13,14]. Amorphous alloy fiber (AAF) is a banded amorphous alloy material [15]. Due to the absence of grain boundaries, dislocations, slip surfaces, and other structures of crystalline materials, amorphous alloy fibers exhibit a series of excellent mechanical and corrosion resistance properties [16,17]. Previous studies have found that [18,19,20,21,22,23,24] the incorporation of amorphous alloy fibers in concrete can effectively improve the mechanical properties of concrete, and they have better corrosion resistance compared with steel fibers. However, the surface of amorphous alloy fiber is characterized by a hydrophobic phenomenon [25], which leads to poor bonding performance between the fiber and cement matrix and cannot give full play to its excellent performance.

Fiber surface modification technology refers to the use of physical or chemical means to improve the surface morphology of fibers or add various functional groups to adjust the wettability between fibers and a matrix, increase the interfacial bonding strength, and further improve the comprehensive performance of composite materials. Physical modification mainly uses mechanical external force, coating viscosity, adsorption, deposition, and other methods to solve the problems of fiber dispersion, fiber surface roughness, and separation of inner and outer layers of fiber bundles in fabrics, including mechanical modification, surface coating modification, plasma modification, and pultrusion process modification. Chemical modification generally occurs by grafting special functional groups on the surface of the fiber so that various substances are combined by covalent bonds, or through the combination of functional groups and matrix. The purpose of improving the interface performance is finally achieved, including the chemical oxidation method, sol–gel method, anodic oxidation method, coupling agent modification, and grafting modification [26]. Silane coupling agent (SCA) is a widely used surface modifier. Zhang Gui et al. [27] used different concentrations of silane coupling agent (SCA) solution to modify the surface of hydrophobic polyethylene fiber, prepared ultra-high-performance concrete (UHPC) with surface-modified PE fiber, and measured its direct tensile strain hardening and cracking behavior. The results showed that, for UHPC with a low water–binder ratio (0.18), 3% SCA solution-modified PE fiber has a better strain-hardening effect, and the multi-crack cracking effect is more significant. The mechanism of modified PE fiber affecting UHPC strain hardening is that the silane coupling agent functional group attached to the surface of PE fiber establishes a strong chemical bonding force with the matrix. Huang Shixin [28] used a silane coupling agent to modify the surface of steel fiber. The optimum surface modification process of steel fiber was determined by the orthogonal test method, and the effect of surface modification of steel fiber on the mechanical properties and durability of ultra-high-performance concrete was investigated. The results showed that the optimum process of modified steel fiber was an alcohol-to-water ratio of 1.3, KH-550 mass concentration of 15%, pH value of 7, and hydrolysis time of 3 h. Under this process condition, UHPC has the best mechanical properties, and surface modification has a certain improvement effect on the durability of UHPC.

At present, the research on the incorporation of modified amorphous alloy fibers into ultra-high-performance concrete is limited. Therefore, in this study, silane coupling agent KH-550 solution was used to modify the surface of amorphous alloy fiber to improve its interfacial bonding performance with a cement matrix, and the effect of modified fiber on the mechanical properties of the ultra-high-performance concrete was further investigated.

## 2. Materials and Methods

### 2.1. Materials

The cement is ordinary Portland cement with P·O52.5. The burning loss of silica fume is 2.43%, the specific surface area is 22,000 m^2^/kg, and the activity index is 118%. The mineral powder is S95-grade mineral powder, the burning loss is 2.3%, the water content is 0.3%, and the density is 2.9 g/cm^3^. Xiamen Aisio standard quartz sand is used as sand. The water-reducing agent adopts the Xika brand 540 P type polycarboxylate superplasticizer produced by Shanghai Chenqi Chemical Technology Co., Ltd. (Shanghai, China), and the water reduction rate is >30%. The water is ordinary tap water, the pH is about 7.5, and the hardness is <200 mg/L. The chemical compositions of cement, silica fume, and mineral powder are shown in Table 1, and the chemical composition and performance parameters of amorphous alloy fiber are shown in Table 2.

### 2.2. Ratio Design

The mix ratios of raw materials in this test were: water–binder ratio was 0.18; sand–binder ratio was 1.1; silica fume–cementitious material ratio was 0.15; and fiber volume contents were 0.4%, 0.8%, and 1.2%, respectively.

### 2.3. Surface Modification of Amorphous Alloy Fibers

**Figure 1:**
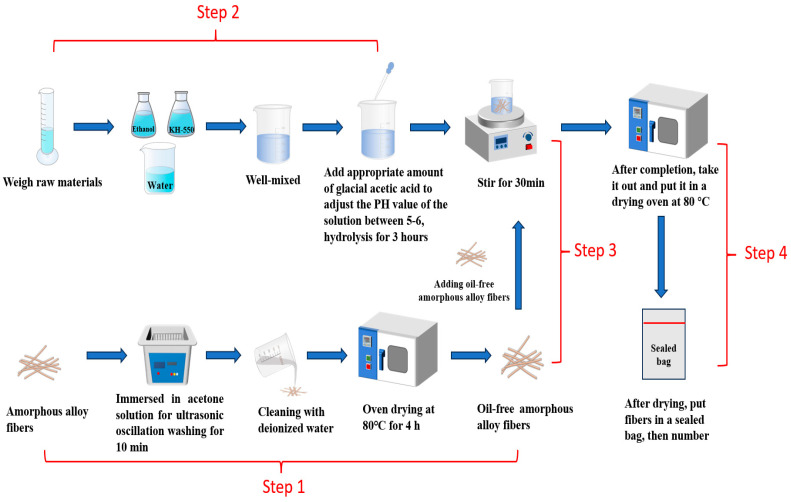
Fiber modification process.

The operation steps of fiber modification were as follows: (1) The fiber was immersed in acetone solution for ultrasonic vibration washing for 10 min, and then washed with deionized water. After washing, it was dried in an oven at 80 °C for 4 h to obtain degreased amorphous alloy fiber. (2) Next, 135 mL of ethanol; 15 mL of water; and 5%, 10%, and 15% KH-550 silane coupling agent were mixed evenly. An appropriate amount of glacial acetic acid was added dropwise to adjust the pH value of the solution between 5–6, and it was then hydrolyzed for 3 h. (3) Amorphous alloy fibers were put into a mixture and stirred for 30 min. (4) After the stirring was completed, the fiber was taken out and dried in an 80 °C drying oven and the dried fibers were put into a sealed bag, numbered, sealed, and spared. Figure 1 shows the fiber modification process.

### 2.4. Preparation and Maintenance of Samples

**Figure 2:**
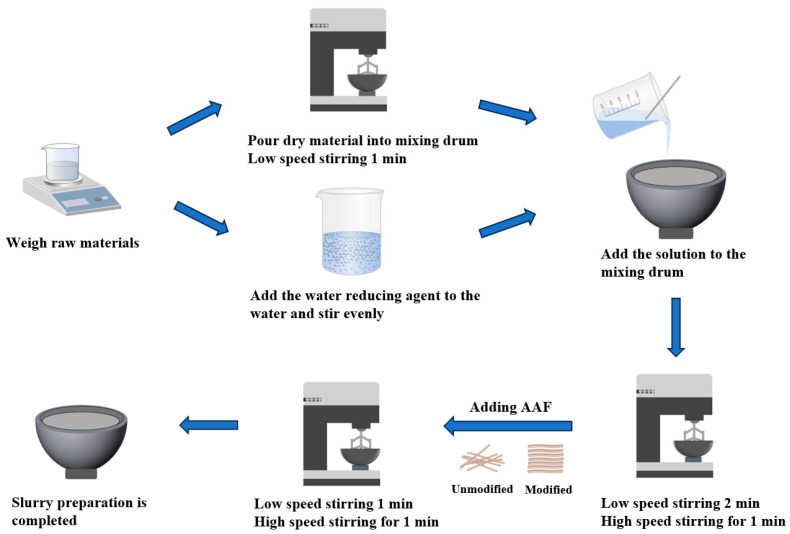
Slurry preparation process.

Figure 2 shows the slurry preparation process.

After the preparation of the slurry, the slurry fluidity test was conducted, and then the slurry was poured according to the corresponding size of the test piece. After the test mold was filled, it was placed on the vibration table for 1 min until it was dense. After the vibration was completed, the mold was scraped flat with a scraping ruler, numbered, and covered with plastic film, then placed in a standard maintenance room for maintenance. After curing for 48 h, the mold was removed and placed in water at (20 ± 1) °C for 28 d.

### 2.5. Test Methods

#### 2.5.1. Fiber Performance Tests

According to the CECS13-2009 standard [29], the fiber–matrix bonding performance was tested. The microcomputer-controlled electronic universal testing machine was used to carry out the test at a loading speed of 0.5 mm/min. The corresponding pull-out load-displacement curve was automatically recorded by the computer.

The scanning electron microscope model S-3400N of the Hitachi Company, Chiyoda, Japan was used to characterize the surface morphology of the specimen. The element composition of the surface of the sample was analyzed by X-ray energy dispersive spectrometer (EDS) of Ultim Extreme, made by Oxford Instrument Technology Co., Ltd. (Shanghai, China). The contact angle tester model, HARKE-SPCA, produced by Beijing HARKE Testing Instrument Factory (Beijing, China), was used for the contact angle test, and the contact angle measurement range was 0–180°. FTIR spectroscopy was used to determine the vibrations and rotations of the different molecular bonds of the main hydration products in the hydration products, and the tests were carried out using an infrared spectrometer manufactured by Thermo Fisher Scientific (Waltham, MA, USA), model Nicolet iS50, with a spectral scanning range of 3500–500 cm^−1^. Laser confocal microscopy uses a laser beam through the illumination pinhole to form a point light source to scan each point of the focal plane in the specimen, and the irradiated point on the specimen is imaged at the detection pinhole, which is received point by point or line by line by the point-multiplier tube or cold electric coupling device behind the detection pinhole to rapidly form a fluorescence image on the screen of the computer monitor. In this study, a laser confocal microscope model, OLS4100 from Olympus Corporation of Tokyo, Japan, was used to characterize the roughness and 3D morphology of the fiber surface. The corrosion resistance of the fiber was characterized by the electrochemical tester chi630e produced by Shanghai Chenhua Instrument Co., Ltd. (Shanghai, China).

#### 2.5.2. UHPC Specimens Mechanical Properties Test

The flexural strength and compressive strength of concrete specimens were tested according to the GB/T 17671-1999 standard [30]. The YAW-300 D microcomputer-controlled automatic compression and bending test machine produced by Jinan New Era Shi Jin Test Instrument Co., Ltd. (Jinan, China) was used. In the flexural strength test, the loading method of 0.2 mm/min displacement control was adopted. The compressive strength test was continuously and uniformly loaded at a loading rate of 2.4 KN/s. The specimens of the direct tensile performance test were dumbbell-shaped specimens. The specimens were taken out after 28 days of standard curing. The electronic universal testing machine was used for the test. The loading method was displacement loading at a rate of 0.6 mm/min.

**Figure 3:**
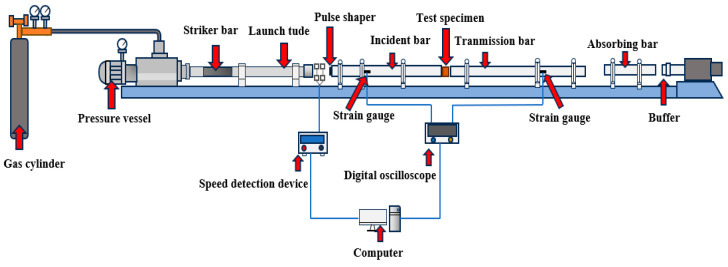
SHPB simplified model.

The dynamic mechanical properties test was carried out using a split Hopkinson pressure bar (SHPB). The device consisted of a pressure bar system composed of an incident bar, transmission bar, and bullet. The data acquisition and analysis system and data processing system are composed of a velocimeter, strain gauge, and strain amplifier. The polished test block is evenly smeared with Vaseline to reduce the error and placed between the incident rod and the transmission rod. The air pressure is used to control the impact speed of the device. When the air pressure reaches the target setting value, the switch is started in order to carry out the dynamic impact compression test. After the test is complete, the relevant data and curves are saved. Figure 3 is a simplified SHPB model.

#### 2.5.3. UHPC Specimens Microstructure Test

The interface area between fiber and cement matrix was investigated by studying the microstructure and structural composition of the interface area. Firstly, the samples cured to the specified age of 28 days were mechanically broken into small pieces, and the samples at the junction of fiber and cement were selected. The samples were soaked in alcohol to terminate their hydration process. Then, the samples were placed in a drying oven for at least 24 h. The samples were taken out and polished with sandpaper so that the maximum radius and thickness did not exceed 2 mm. Finally, the prepared sample was polished for surface gold spraying, and the microstructure of the fiber–cement matrix interface area was characterized by scanning electron microscopy (S-3400N).

## 3. Results and Discussion

### 3.1. Fiber Microstructure and Contact Angle

**Figure 4:**
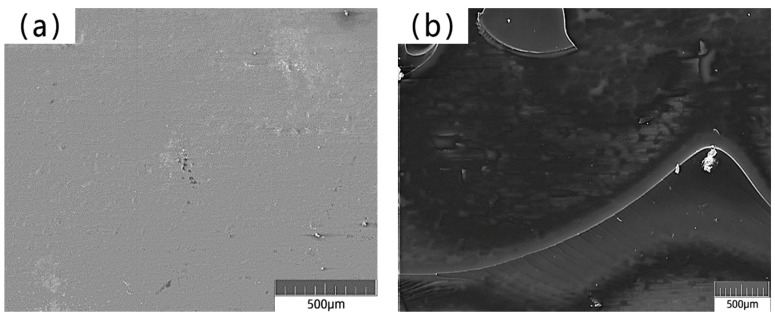

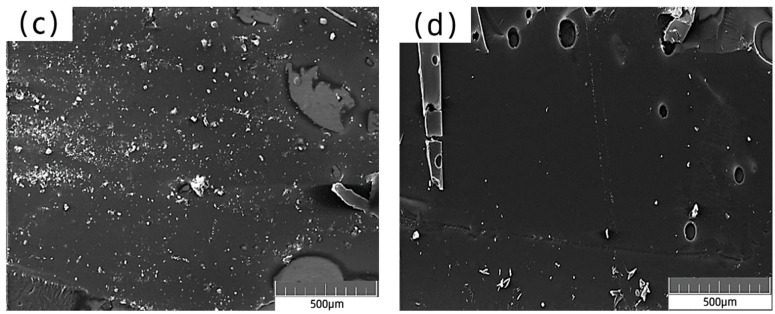
Surface morphology of amorphous alloy fibers modified by different concentrations of KH-550 solution. (**a**) Unmodified; (**b**) 5%; (**c**) 10%; (**d**) 15%.

The microstructures of the fibers were observed using a S-3400 N scanning electron microscope, and the change in the microstructure of the fiber before and after modification was obtained, as shown in Figure 4. The surface of the unmodified amorphous alloy fiber was smooth, and the surface of the modified amorphous alloy fiber was covered with a light grey silane film. With the increase in the concentration of the modified solution, it could be seen that the surface of the fiber was smooth from the surface to the formation of an uneven and thin silane film, and there were more voids. Then, the modified layer was gradually thickened, and finally, the surface was covered with a thick silane film.

The surface roughness and three-dimensional morphology of the amorphous alloy fibers before and after modification were characterized by laser confocal microscopy.

**Figure 5:**
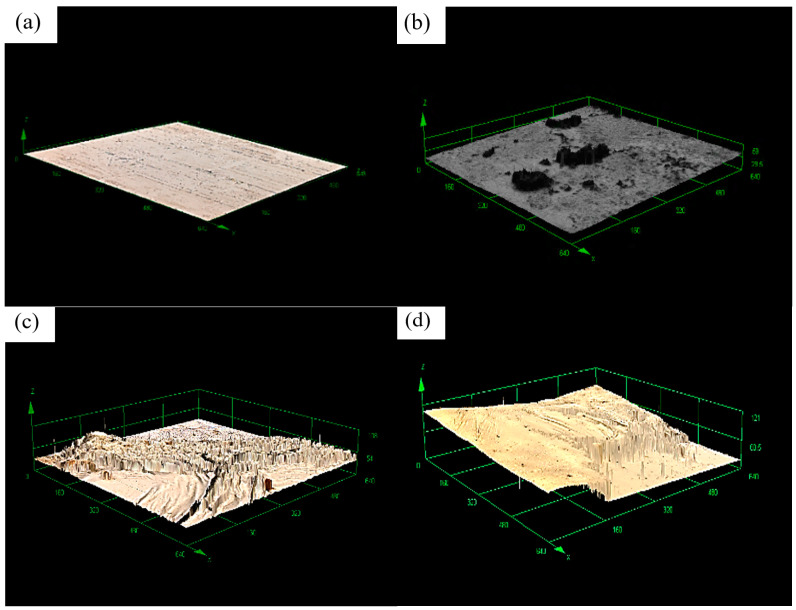
Three-dimensional map of amorphous alloy fiber surface modified by different concentrations of silane coupling agent KH-550 solution. (**a**) Unmodified; (**b**) 5%; (**c**) 10%; (**d**) 15%.

From Figure 5 and Table 3, we can see a trend that the roughness increased with the increase in concentration of the modified solution; the maximum roughness reached 3.85.

**Figure 6:**
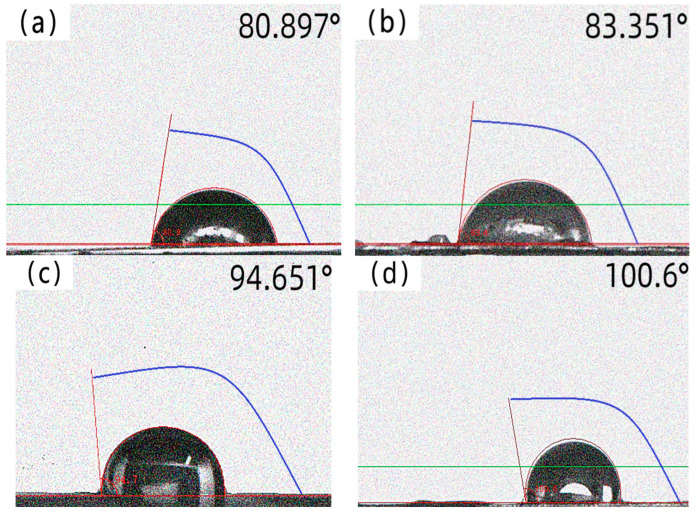
The contact angle of amorphous alloy fiber modified by different concentrations of silane coupling agent KH-550 solution: (**a**) unmodified; (**b**) 5%; (**c**) 10%; (**d**) 15%.

The contact angle is an important index to assess the surface wettability of materials; the smaller the contact angle, the better its wettability performance. The contact angle of amorphous alloy fiber before and after modification were tested by a contact angle meter. The results are shown in Figure 6, the contact angles of amorphous alloy fiber modified with silane coupling agent KH-550 solution were all increased to different degrees.

**Figure 7:**
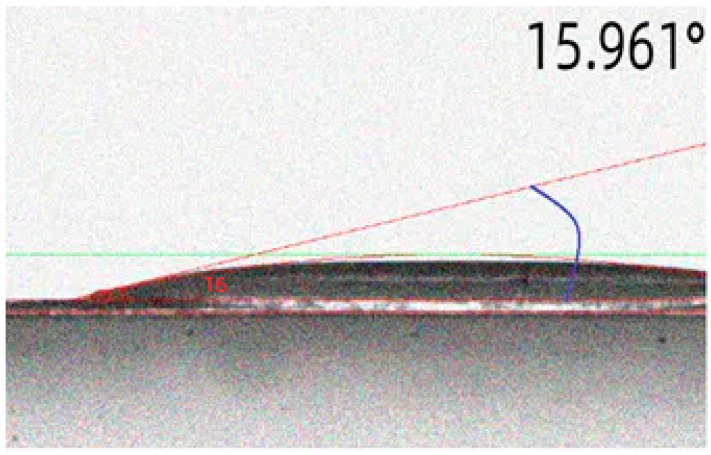
Contact angle of modified amorphous alloy fiber after long-term immersion in water.

Compared with the unmodified amorphous alloy fiber, the surface contact angle of the amorphous alloy fiber modified by a 15% concentration increased by 24.4%, reaching 100.6°. This may have been due to the effect of the silane coupling agent. A hydrophobic film was formed on the surface of the amorphous alloy fiber, so that the contact angle began to increase [31,32,33,34,35]. After long-term immersion, the contact angle became smaller, as shown in Figure 7. This may be due to the fact that the silane coupling agent molecules gradually hydrolyzed to form a silane alcohol structure under long-term solution immersion, and finally showed better hydrophilicity [36,37,38,39,40].

### 3.2. Fiber Surface Element Content and Functional Groups

**Figure 8:**
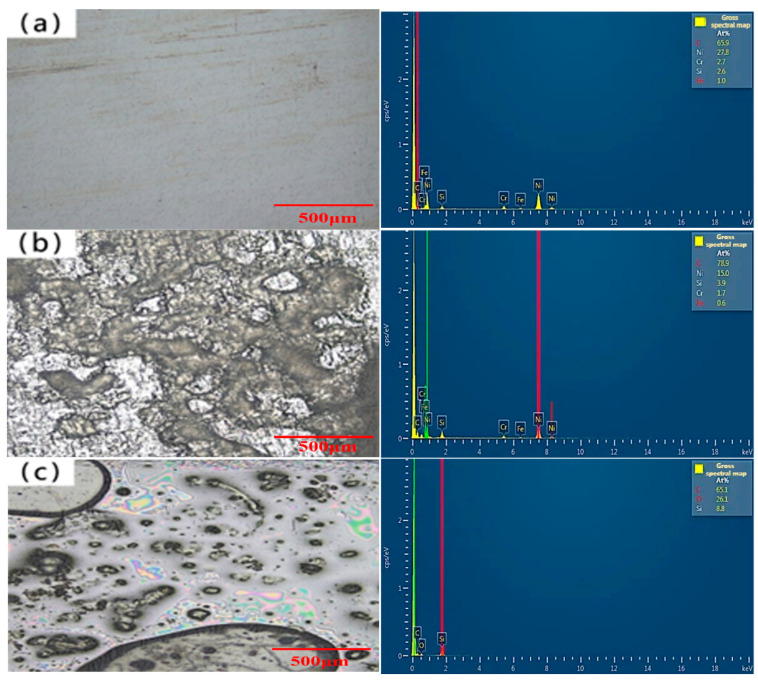

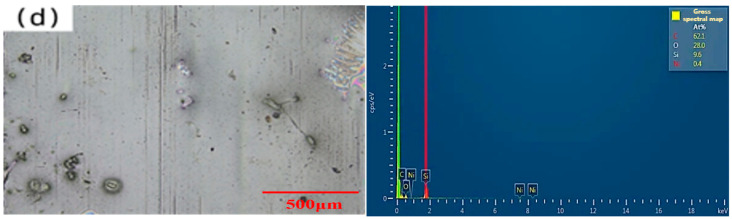
The chemical composition of the surface of amorphous alloy fiber before and after modification: (**a**) unmodified; (**b**) 5%; (**c**) 10%; (**d**) 15%.

EDS scanning of the surface of the amorphous alloy fiber modified with different concentrations of silane coupling agent KH-550 solution was performed, and the results are shown in Figure 8.

The surface of the unmodified amorphous alloy fiber was smooth, and the main elements were C, Ni, Si, and Cr. The surface of the amorphous alloy fiber modified by the silane coupling agent produced the O element, and the contents of Si and O increased with the increase in the concentration of the silane coupling agent KH-550 solution. This shows that the hydrolyzed silane coupling agent product was adsorbed on the surface of the amorphous alloy fiber after the modification of the amorphous alloy fiber. A stable silane film was formed, so the contents of Si and O elements on the surface changed.

**Figure 9:**
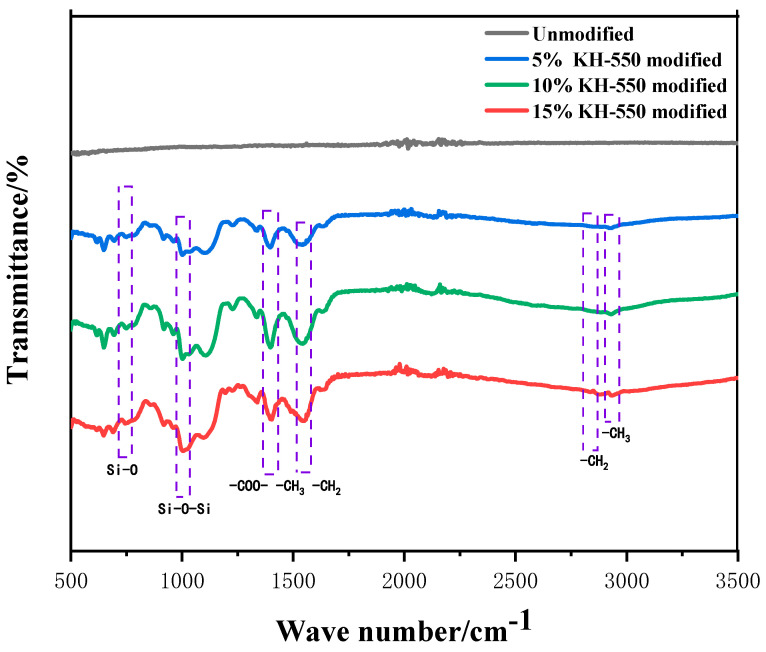
Infrared spectra of amorphous alloy fibers modified by different concentrations of silane coupling agent KH-550 solution.

The surface functional groups of amorphous alloy fibers before and after modification were tested using a Fourier transform infrared spectrometer. The test results are shown in Figure 9.

Six obvious new peaks can be observed between 700 and 1600 cm^−1^: the symmetrical stretching vibration peaks of Si-O bond at 744 cm^−1^; the symmetrical stretching vibration peak of Si-O-Si bond at 1004 cm^−1^; the peaks at about 1403 cm^−1^ and 1544 cm^−1^, which were also significantly enhanced, which may have been caused by the superposition of the characteristic peaks of -COO-, -CH_3_, and -CH_2_; and the stretching vibration absorption peaks of KH-550 silane coupling agent-CH_3_ and-CH_2_, which appeared near 2940 cm^−1^ and 2850 cm^−1^. Combined with previous studies [41], the generation of significant peaks such as Si-O-Si shows that, after modification, an outer silane film with Si-O-Si interpenetrating network structure formed on the surface of the amorphous alloy fiber.

### 3.3. Analysis of Fiber Corrosion Resistance

**Figure 10:**
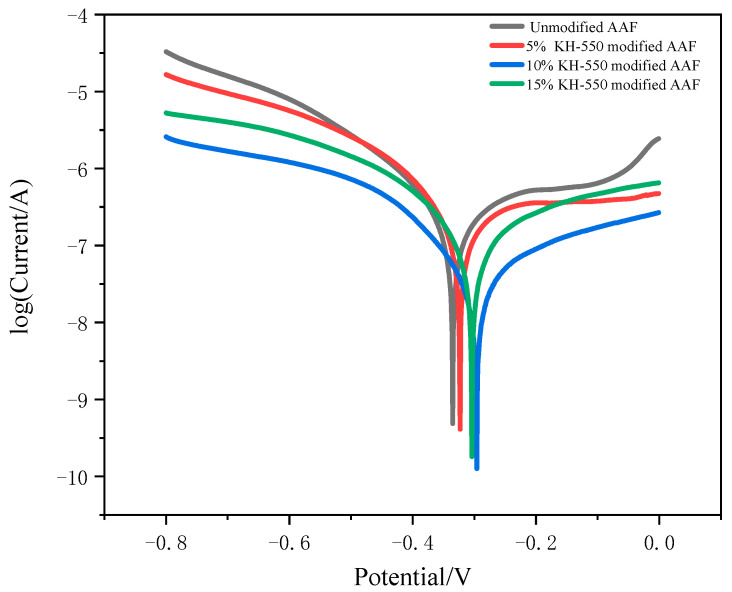
Polarization curves of amorphous alloy fibers modified by different concentrations of silane coupling agent KH-550 solution in 3.5% NaCl solution.

A Chenhua chi630e electrochemical workstation was used to test the potentiodynamic polarization curves of different samples in 3.5% NaCl solution. Figure 10 shows the polarization curve of amorphous alloy fiber modified by different concentrations of silane coupling agent KH-550 in 3.5% NaCl solution. It can be seen that, with the increase in the concentration of silane coupling agent KH-550, the corrosion resistance of the amorphous alloy fiber also increased. This was because the modification of amorphous alloy fiber formed a layer of anti-corrosion silane film on the surface, which reduced the corrosion degree of amorphous alloy fiber.

### 3.4. Bonding Properties of Fiber Analysis

**Figure 11:**
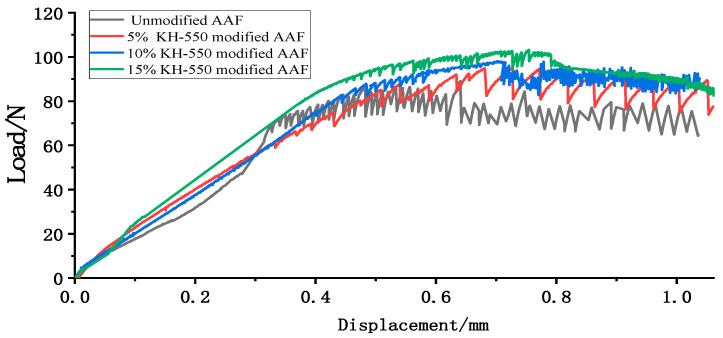
Load–displacement curve of amorphous alloy fiber pull-out test.

Figure 11 shows the drawing load–displacement curve of amorphous alloy fibers modified by silane coupling agent KH-550 solution with different concentrations.

**Figure 12:**
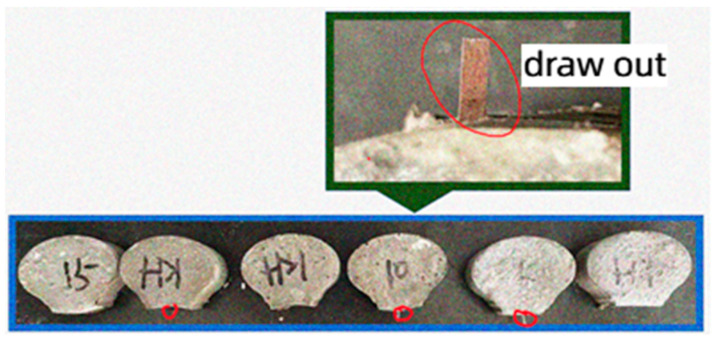
Pull-out failure mode of modified amorphous alloy fiber.

With the increase in the silane coupling agent concentration, the force required for fiber to pull out from the matrix was also greater. The peak load of the fiber modified by 15% silane coupling agent KH-550 solution was 103.22 N, which was 15.9% higher than that of the unmodified fiber. The failure mode of amorphous alloy fiber modified by three different concentrations of silane coupling agent KH-550 solution was the mode of fiber pulling out from the matrix, as shown in Figure 12.

There were obvious scratches on the surface of the drawn amorphous alloy fiber, and tiny mortar particles were attached. The main reason is that the silane coupling agent KH-550 hydrolyzes to generate Si-OH. One end is condensed with the surface of the hydroxylated amorphous alloy fiber so that the fiber and the silane coupling agent are effectively and stably “connected”. A layer of silane film is formed on the surface of the fiber, which enhances the activity and corrosion resistance of the fiber, while the other end reacts with the hydroxyl group on the surface of the hydrated calcium silicate of the cement hydration product so that the fiber is more closely combined with the cement mortar matrix, and the bonding between the fiber and the mortar is improved.

Table 4 shows the results of the fiber pull-out test. The fiber modified by 15% solution had the maximum bond strength and pull-out energy, which were 3.29 MPa and 74.61 N mm, respectively.

By comparing the surface morphology and chemical composition of fibers modified by different concentrations of silane coupling agent KH-550 solution, it was found that when the concentration was low, the surface of the fiber was only partially attached to the silane coupling agent, and the adsorption rate was low. When the concentration was 15%, the silane film was uniform and thick, which made the interface mechanical properties between the fiber and the cement matrix optimal. Therefore, the modified fibers required for the subsequent test of UHPC were all amorphous alloy fibers modified by 15% silane coupling agent KH-550 solution.

### 3.5. Compressive Strength and Flexural Strength Analysis

**Figure 13:**
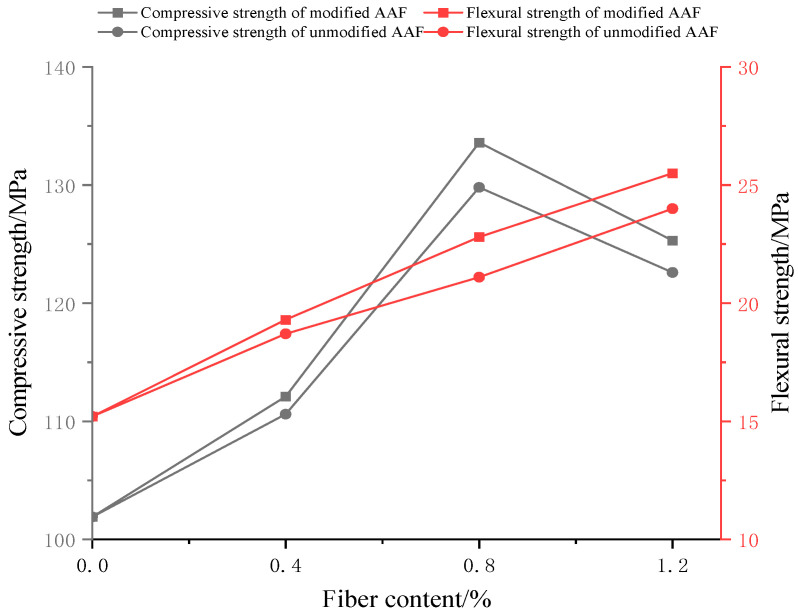
Effect of KH-550 modified amorphous alloy fiber on flexural and compressive strength of UHPC.

In order to investigate the effect of modified fibers on the mechanical properties of UHPC, compressive strength and flexural strength tests were carried out on the specimens. The results are shown in Figure 13. Under the same fiber content, the mechanical properties of modified amorphous alloy fiber UHPC were better than those of unmodified amorphous alloy fiber UHPC.

With the increase in fiber content, the flexural strength increased gradually. When the fiber content was 1.2%, the flexural strength of the modified fiber group and the unmodified fiber group reached 25.5 MPa and 24 MPa, respectively. The increase in flexural strength was because the modified amorphous alloy fiber was more closely combined with cement mortar. When an appropriate amount of fiber was added, the test block needed more energy to peel off the external force fiber from the matrix.

With the increase in fiber content, the compressive strength increased first and then decreased. When the fiber content was 0.8%, the compressive strength of the modified fiber group and the unmodified fiber group reached 133.6 MPa and 129.8 MPa, respectively. The reason for the increase in compressive strength is that the surface of the amorphous alloy fiber modified by silane coupling agent KH-550 was covered with a layer of silane film, which became a silanol structure under the infiltration of water molecules for a long time. It can react with the hydroxyl group in the hydrated calcium silicate of cement hydration product to form Si-O-Si, which makes the fiber and cement matrix have better overall bonding, thus increasing the compressive strength of UHPC. When the fiber content is too much, the compressive strength will decrease, which may be due to the agglomeration of fibers [42,43,44,45].

### 3.6. Direct Tensile Properties Analysis

**Figure 14:**
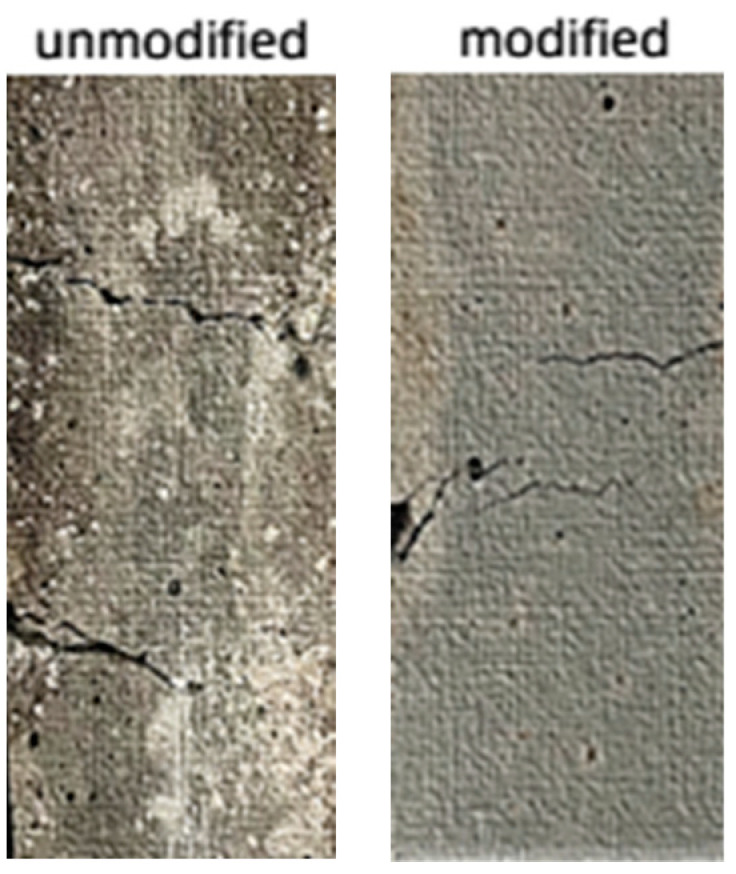
Failure mode of direct tensile specimen of amorphous alloy fiber UHPC.

Figure 14 shows the failure mode of the direct tensile test specimens of the unmodified amorphous alloy fiber group and the modified amorphous alloy fiber group at 1.2% fiber content.

The surface of the specimen with the unmodified amorphous alloy fiber group produced two large cracks and penetrated the cross section. The number of cracks in the modified amorphous alloy fiber group was more than that in the unmodified group, and many tiny cracks appeared. This was due to the fact that the surface modification treatment of the amorphous alloy fiber improved the performance of the fiber–cement matrix interface, giving full play to the excellent mechanical properties of the amorphous alloy fiber itself so that the specimen could be better transmitted between fiber and cement matrix when subjected to external force.

It can be seen from the data in Table 5 that the initial crack strength, tensile strength, and ultimate strain increase with the increase in fiber content indicate that the modified amorphous alloy fiber can effectively improve the tensile strength of the specimen.

### 3.7. Dynamic Mechanical Properties Analysis

**Figure 15:**
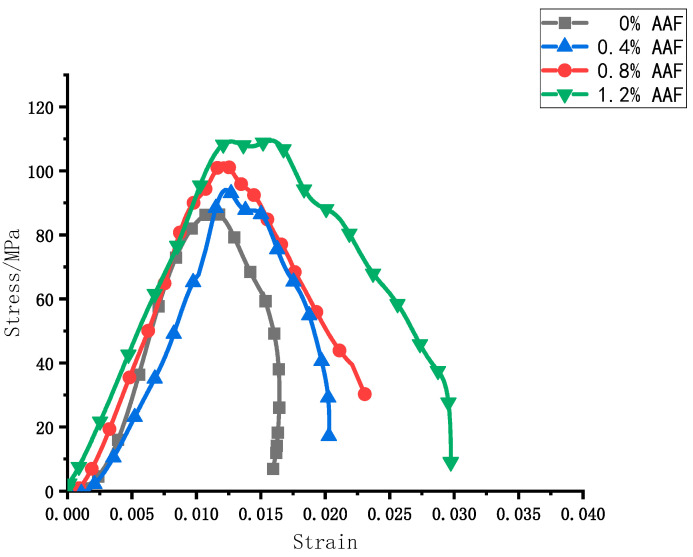
Dynamic stress–strain curves of UHPC with different volume fractions of amorphous alloy fiber.

The dynamic mechanical properties of UHPC were studied by using the split Hopkinson pressure bar device. Figure 15 shows the dynamic stress–strain curve of UHPC specimens with different amorphous alloy fiber contents under unmodified conditions.

According to the image, under the same impact pressure of 0.3 MPa, the dynamic stress–strain curves of specimens with different fiber contents were roughly the same. In the whole process of stress and deformation of the specimen, because of the pores and microcracks existing in the concrete specimen itself, it is subjected to the combined effect of strain hardening (low strain rate) and damage softening (high strain rate) during the matrix compression process. Firstly, the curve is on the rise, that is, the stress increases with the increase in strain and shows a linear relationship before a certain stress value. With the increase in load, cracks inside the specimen continue to crack and expand outward. At this time, the stress and strain change into a non-linear relationship. When the load reaches the peak, the amorphous alloy fiber group and the non-doped fiber group show different performances. The fiber group has a longer stress platform. The stress platform becomes more obvious with the increase in the amorphous alloy fiber content. This is because the addition of amorphous alloy fibers improves the ability of the matrix to resist crack propagation.

**Figure 16:**
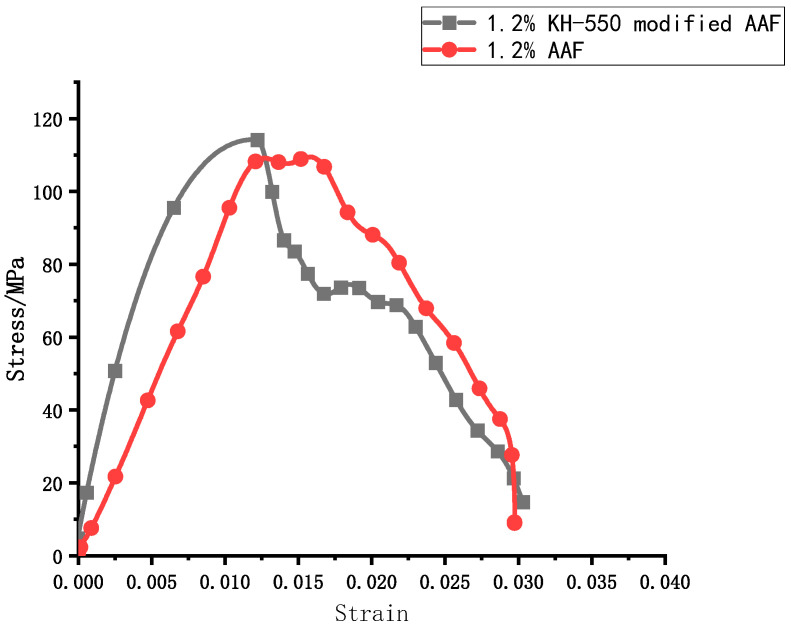
UHPC stress–true strain curve of amorphous alloy fiber.

At 1.2% amorphous alloy fiber doping, the peak stress of UHPC was the largest. Based on this fiber doping, the UHPC doped with modified amorphous alloy fibers was tested under the same impact air pressure of 0.3 MPa, and finally, the stress–strain curve under this doping was obtained, as shown in Figure 16.

Table 6 shows the dynamic mechanical test results of UHPC with amorphous alloy fibers. When the content of unmodified fiber was between 0 and 1.2%, the peak stress of the specimen increased with the increase in fiber content, and reached the maximum when the content was 1.2%.

The peak stress of the modified amorphous alloy fiber was 4.3% higher than that before modification. This is because the modification of amorphous alloy fiber can greatly improve the interfacial compatibility and bonding strength between fiber and cement matrix and improve the impact resistance of concrete materials.

**Figure 17:**
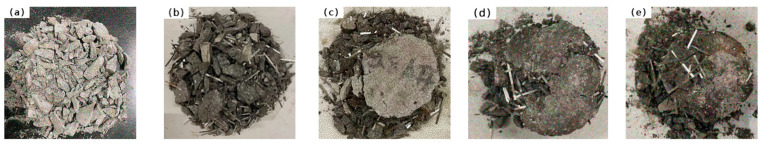
Impact compression failure morphology of amorphous alloy fiber UHPC before and after modification: (**a**) no fibers; (**b**) 0.4% AAF; (**c**) 0.8%AAF; (**d**) 1.2% AAF; (**e**) 1.2% KH-550 AAF.

Figure 17 shows the morphology of post-impact damage of UHPC specimens with different amorphous alloy fiber contents before and after modification.

Figure 17a shows a broken ring diagram of the unadulterated fiber cement mortar specimen. The specimens present uniform sizes of fragmentation damage; there were a large number of sharp particles; the damage produced by the cement broken small particles splashed out; and the damage state was more serious, in line with the brittle damage characteristics. After mixing amorphous alloy fibers, as shown in Figure 17b–d, the broken state of the specimen was improved and there were more cracks, but not broken state, the fibers were the cement mortar as a whole bridged together. The degree of specimen damage splash was greatly reduced, and the brittleness of the cement mortar specimen was improved. And the integrity of the specimens was higher when the amorphous alloy fiber doping was 1.2%. Figure 17e shows the damage state of the cement mortar specimens with modified amorphous alloy fibers under the same impact air pressure, and by comparing with Figure 17d, it can be found that after the cement mortar specimen mixed with the modified amorphous alloy fibers was destroyed, the splashed cement particles were fewer, the cracks were smaller than before the modification, and the matrix integrity was better. The addition of amorphous alloy fiber can effectively improve the impact resistance of UHPC; the impact resistance of UHPC with modified fiber was better than that of UHPC with unmodified fiber. This was because the surface roughness of the modified fiber increased, which increased the friction force, and the interfacial bonding strength between modified fiber and cement matrix was also higher. In previous studies [46,47,48], there have been similar conclusions.

### 3.8. Interface Transition Zone Analysis

**Figure 18:**
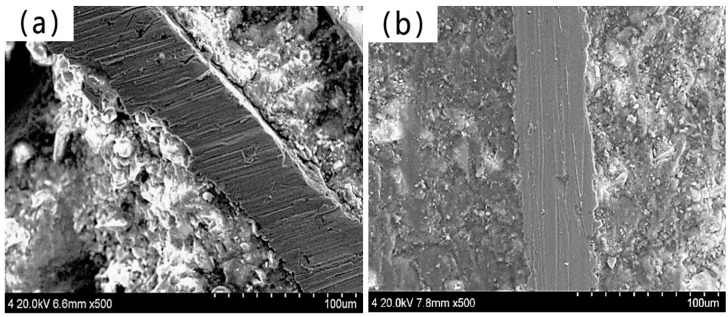
SEM image of interfacial transition zone between amorphous alloy fiber and mortar: (**a**) Unmodified amorphous alloy fiber and mortar interface transition zone; (**b**) KH-550 modified amorphous alloy fiber and mortar interface transition zone.

The interfacial transition zone was observed and analyzed using a scanning electron microscope. Figure 18a shows the interfacial transition zone between the unmodified fiber and the cement matrix.

Figure 18b shows the interfacial transition zone between the modified fiber and cement matrix by silane coupling agent KH-550 solution. The gap between the interface of unmodified amorphous alloy fiber and cement matrix is large, the bonding force is weak, and the structure of interface transition zone is loose. The above phenomenon was mainly due to the smooth surface of the amorphous alloy fiber and the low surface energy, resulting in poor bonding between the fiber and the cement matrix. When subjected to external force, the amorphous alloy fiber easily slips out of the cement matrix, affecting its mechanical properties. The surface of the modified amorphous alloy fiber adsorbs the silane coating, which makes the surface of the amorphous alloy fiber become wrinkled and rough and have higher activity. When it is added to the cement mortar, the hydroxyl group on the surface of the cement hydration product C-S-H gel condenses with the silane molecule Ni-Si-OH on the surface of the amorphous alloy fiber, so the fiber–matrix bonding effect is better. The interface between the surface of the modified amorphous alloy fiber and the cement matrix is closer than that before the modification.

### 3.9. Interface Model Analysis

**Figure 19:**
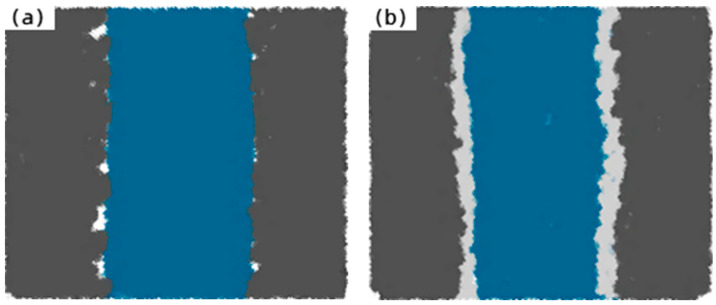
Interface model between different modified amorphous alloy fibers and cement matrix: (**a**) The interface model between unmodified amorphous alloy fiber and cement matrix; (**b**) The interface model between KH-550 modified amorphous alloy fiber and cement matrix.

The surface morphology of the fiber is an important factor affecting the degree of interface bonding between the fiber and the matrix. Figure 19a shows the interface model between the unmodified fiber and the cement matrix.

Figure 19b shows the interface model between the modified fiber and the cement matrix by the silane coupling agent KH-550 solution. It can be found that the surface roughness of the modified amorphous alloy fiber increased, the anchoring force increased, the fiber surface energy improved, the compatibility between the amorphous alloy fiber and the cement matrix improved, and the contact area with the cement matrix increased so that the interface between the fiber and the cement was firm. Therefore, the mechanical properties of the samples with modified amorphous alloy fibers were significantly higher than those of the samples with unmodified amorphous alloy fibers. By increasing the interfacial bonding strength, the sample with modified amorphous alloy fibers was cracked and the stress transferred from the cement matrix to the fiber itself through the interfacial transition zone was greater, thereby preventing the expansion of cracks. Therefore, the toughening effect of the sample with modified amorphous alloy fiber was higher than that of the sample with unmodified amorphous alloy fiber.

## 4. Conclusions

In this study, silane coupling agent KH-550 solution was used to modify amorphous alloy fiber, the modification effect was analyzed, and its influence on the mechanical properties of ultra-high-performance concrete was further investigated. Through microstructure analysis, the influence mechanism was explored, and the following conclusions were obtained:

1. The 15% silane coupling agent KH-550 solution had the best modification effect on the fiber. At this time, the interfacial bonding strength between the fiber and the cement matrix reached 3.29 MPa, and the roughness reached 3.85. Compared with the unmodified fiber, it increased by 15.8% and 6316.7%, respectively. The contact angle was reduced to 15.961°, which was 80.3% lower than that of the unmodified fiber.
2. The modified amorphous alloy fiber could effectively improve the mechanical properties of UHPC. When the fiber content was 0.8%, the compressive strength reached the maximum, which was 133.6 MPa. When the fiber content was 1.2%, the flexural strength and tensile strength reached the maximum, which were 25.5 MPa and 8.32 MPa, respectively.
3. UHPC with modified fibers was compared with UHPC with unmodified fibers. The most significant increases in compressive strength and flexural strength were observed at a 0.8% fiber content, with increases of 2.9% and 8.1%, respectively. The most significant increase in tensile strength was 10.9% at a 1.2% fiber content.
4. When the fiber content was 1.2%, the peak stress of UHPC with modified amorphous alloy fiber reached 114.26 MPa, which was 4.3% higher than that of UHPC with the same amount of unmodified amorphous alloy fiber and 31.3% higher than that of UHPC without fibers.

## Figures and Tables

**Table 1:** Chemical composition of raw materials (%).

	CaO	SiO_2_	Al_2_O_3_	Fe_2_O_3_	MgO	SO_3_
Cement	64.4	24	4.75	3.45	1.99	1.32
Silica fume	0.23	96.82	0.21	-	-	-
Mineral powder	35.58	36.1	16.32	-	11.32	-

**Table 2:** Chemical composition and performance parameters of amorphous alloy.

Density (g/cm^3^)	Length (mm)	Length to Diameter Ratio	Tensile Strength/MPa	Acid-Alkali Resistance	Conductivity	Elementary Composition
7.2	30	15	1400	High	High	C, Si, Ni, Cr Fe, B, P

**Table 3:** Results of roughness measurements.

Sample	Roughness
Unmodified	0.06
5% KH-550 modified	1.42
10% KH-550 modified	3.36
15% KH-550 modified	3.85

**Table 4:** Fiber pull-out test results.

Silane Coupling Agent KH-550 Concentration/%	Peak Load /N	Peak Displacement/mm	Bond Strength /MPa	Pull-Out Energy /N·mm
0	89.04	0.64	2.84	60.35
5	94.84	0.68	3.02	66.03
10	98.13	0.70	3.13	69.04
15	103.22	0.75	3.29	74.61

**Table 5:** Test results of UHPC direct tensile test with volume fraction of amorphous alloy fiber.

	Volume Fraction of AAF/%	Initial Crack Strength/MPa	Tensile Strength /MPa	Ultimate Strain
Unmodified AAF	0	4.47	4.47	0.39
	0.4	4.58	5.17	0.58
	0.8	6.3	6.53	0.79
	1.2	6.29	7.5	0.92
Modified AAF	0.4	4.56	5.48	0.64
	0.8	5.87	6.89	0.74
	1.2	5.96	8.32	0.97

**Table 6:** Dynamic mechanical test results of amorphous alloy fiber UHPC.

Modification Methods	AAF Content /%	Average Strain Rate/s^−1^	Peak Stress /MPa	Peak Strain
Unmodified	0	90.64	87.04	0.011
	0.4	97.89	94.04	0.012
	0.8	103.21	102.35	0.012
	1.2	110.56	109.52	0.016
KH-550 modified	1.2	114.61	114.26	0.012

## Data Availability

The original contributions presented in the study are included in the article, further inquiries can be directed to the corresponding author.
